# Environmental benefits of reintroducing reusable drapes and gowns in the operating room

**DOI:** 10.1093/bjsopen/zrag139

**Published:** 2026-09-21

**Authors:** Florine I de Haes, Meis Uijttewaal, Nicolaas H Sperna Weiland, Martijn L M Broeren, Dorien A Salentijn

**Affiliations:** Centre for Sustainable Healthcare, Amsterdam UMC, Amsterdam, the Netherlands; Epidemiology and Data Science, Amsterdam UMC, Amsterdam, the Netherlands; CE Delft, Delft, the Netherlands; Epidemiology and Data Science, Amsterdam UMC, Amsterdam, the Netherlands; CE Delft, Delft, the Netherlands; Centre for Sustainable Healthcare, Amsterdam UMC, Amsterdam, the Netherlands; Department of Anesthesiology, Amsterdam UMC, University of Amsterdam, Amsterdam, the Netherlands; Epidemiology and Data Science, Amsterdam UMC, Amsterdam, the Netherlands; CE Delft, Delft, the Netherlands; Centre for Sustainable Healthcare, Amsterdam UMC, Amsterdam, the Netherlands; Epidemiology and Data Science, Amsterdam UMC, Amsterdam, the Netherlands; Department of Radiology and Nuclear Medicine, Amsterdam UMC, University of Amsterdam, Amsterdam, the Netherlands

**Keywords:** Surgical trays, procedure trays, disposable, life cycle assessment, sustainable healthcare

## Abstract

**Background:**

The healthcare sector contributes significantly to environmental pollution, which adversely affects human health. Operating rooms are a major source of this impact, partially due to the routine use of disposable materials prepacked in surgical trays. The main objective of this study was to compare the environmental impacts of fully disposable surgical trays with those of hybrid surgical trays, which replace disposable drapes and gowns with reusable ones.

**Methods:**

This multicentre study included surgical trays from three types of surgeries: gynaecological laparoscopy surgery, extremity surgery, and caesarean section. This selection captures a broad range of surgical workflows and instrument requirements, enabling the development of an average tray composition and enhancing the generalizability of the findings across various surgical specialties. The hybrid trays, including reusable drapes and gowns, were developed and tested specifically for this study. A life cycle assessment was conducted according to ISO 14040/44 standards to compare the environmental impacts of the hybrid and disposable trays across 18 impact categories.

**Results:**

Hybrid trays demonstrated lower environmental impacts than disposable trays across almost all impact categories. For climate change, hybrid trays reduced impact by 40–51%. In disposable trays, drapes and gowns contributed to approximately 69% of the total climate change impact, whereas in hybrid trays this contribution decreased to 38%, primarily because the production and end-of-life impacts of reusable drapes and gowns are distributed over multiple uses.

**Conclusion:**

These results provide a robust evidence base to inform and support a broader European Union-wide transition to hybrid trays, incorporating reusable drapes and gowns, in operating rooms.

## Teaser

Hybrid trays, including reusable drapes and gowns, consistently demonstrate lower environmental impacts across multiple indicators (for example, marine ecotoxicity and fossil resource scarcity), including a 40–51% reduction in climate change impact compared with a fully disposable tray. These findings highlight the environmental benefits of incorporating reusable materials in healthcare and provide evidence to support a European Union-wide transition towards reusable textiles in operating rooms.

## Introduction

Globally, the healthcare sector is estimated to be responsible for 5% of total greenhouse gas emissions, along with significant contributions to air pollution, water use, and waste production^1^. Although the sector’s primary goal is to protect and promote human health, it is also a major contributor to the climate crisis, which is the greatest health threat of the 21st century^2^.

Within the hospital setting, the operating room is one of the hotspots for environmental impact; it accounts for a high share of energy consumption and waste production^3,4^. A key contributor to this waste production is the routine use of disposable surgical materials, often bundled in prepacked surgical trays. These trays frequently contain more items than are used in practice^5,6^. As a result, unused items are discarded, leading to unnecessary resource consumption and waste generation.

To advance more sustainable surgical care, it is important to identify sources of avoidable environmental impact and explore strategies for improvement. This study uses life cycle assessment (LCA) to quantify the environmental impact of surgical trays, covering the entire life cycle from the production of raw materials, usage, and ultimately their end-of-life disposal. In addition, LCA identifies environmental hotspots within these surgical trays, enabling the prioritization of components with the greatest potential for impact reduction. In this study, two tray configurations were compared: one consisting entirely of disposable products, including drapes and gowns (the disposable tray), and the other one in which the drapes and gowns are reusable (the hybrid tray). This comparison builds on previous evidence that reusable textiles can substantially reduce environmental impact compared with single-use alternatives^7,8^. The use of reusable drapes and gowns is not necessarily new, because it was the standard practice before the widespread adoption of disposable textiles only in the late 20th century^8^.

This study is part of the larger multicentre rEUsable project, which aims to promote more sustainable surgical practices across the European Union^9^. For this study, hybrid trays were specifically developed to investigate and evaluate their potential benefits. Complementary studies within the project will assess the economic, organizational, and behavioural aspects of implementing hybrid trays, together contributing to a comprehensive understanding of how such interventions can support the transition towards more sustainable surgical care throughout Europe.

## Methods

This study was conducted in the Netherlands between January 2025 and May 2026. The procedure trays analysed were obtained from three hospitals: Amsterdam UMC (caesarean section tray), Onze Lieve Vrouwe Gasthuis (gynaecological laparoscopy tray), and Leiden University Medical Center (extremity surgery tray). The rEUsable project has been reviewed and approved by the local Medical Ethics Research Committee (METC) as not being subject to Medical Research Involving Human Subjects Act regulations or written informed consent (METC: 2024-0477 Project rEUsable).

### Life cycle assessment

LCA was used to quantify the environmental impacts of hybrid and disposable trays in accordance with ISO 14040/44. LCA is a standardized method to determine the environmental impacts of products or services throughout their entire life cycle^10^. A Microsoft^®^ Excel (Microsoft, Redmond, WA, USA)-based LCA model was developed that includes all products on both trays and calculates their associated environmental impacts. The model is designed to be adaptable within the rEUsable project, enabling modification of key parameters, such as country-specific data or product quantities, to support in-depth analyses. Throughout this article, the tool is referred to as the rEUsable LCA model.

### Goal and functional units

The goal of this study was to determine and compare the cradle-to-grave environmental impacts of disposable and hybrid surgical trays used for three types of surgery. The hybrid tray contains reusable drapes and gowns, which are washed, sterilized, and reused, whereas the disposable tray contains conventional single-use drapes and gowns. Other disposable products, such as syringes, needles, and bowls, are the same on both trays and are referred to as standard disposable products. Furthermore, both trays require packaging, which is referred to as additional disposable packaging. Although hybrid trays reduce the primary production and disposal of drapes and gowns, the drapes and gowns do need to be collected, transported, washed, and sterilized before they can be reused in a procedure tray.

The following functional units are used for the assessment: a procedure tray required for one Caesarean section (C-section) surgery; a procedure tray required for one gynaecological laparoscopy surgery; and a procedure tray required for one extremity surgery. In addition to calculating the environmental impact of each of these trays, the average environmental impact of the three procedure trays is calculated for the disposable and hybrid variants. This provides an estimate of the impact of an average tray.

It is important to note that the composition of procedure trays may vary slightly between hospitals for a given surgery. The disposable trays analysed in this study are based on real-world configurations used in two academic hospitals and one peripheral hospital in the Netherlands. The hybrid procedure trays maintain identical compositions, with the exception that disposable drapes and gowns are replaced by their reusable counterparts. These are functionally equivalent to the disposable trays. Current evidence and international guidelines have also declared no difference in infection risk between reusable and disposable drapes and gowns^11,12^. The hybrid trays are clinically tested in the European Union-funded rEUsable project.

### Surgical trays studied

The surgical trays consist of various products, as listed in *Table 1*. Each tray includes different types and sizes of drapes. The standard disposable products are the same on both trays, but the hybrid tray requires additional packaging for the reusable drapes and gowns, which is why the number for additional disposable packaging is higher for the hybrid tray. A more detailed overview of the products in each tray is provided in *Supplementary methods S1 Table S1–Table S9*.

**Table 1:** Overview of the number and weight of drapes and gowns and (standard) disposable products on each surgery tray

	Caesarean section		Gynaecological laparoscopy		Extremity surgery	
**Drapes and gowns (different products on both trays)**						
	Disposable	Hybrid	Disposable	Hybrid	Disposable	Hybrid
Drapes (*n*)	6	6	4	4	7	7
Surgical gowns (*n*)	2	2	3	3	3	3
Weight (kg)	1.7	3.9	1.5	5.1	2.0	5.4
**Standard disposable products (same products on both trays)**						
Tubes (*n*)	3		2		2	
Bandage/gauze^A^ (*n*)	5		1		2	
Surgical blades (*n*)	3		1		3	
Plastic bowls (*n*)	4		3		7	
Bags (*n*)	3		4		1	
Other components (*n*)	7		3		6	
Weight (kg)	0.9		0.7		0.6	
**Disposable products (different products on both trays)**						
	Disposable	Hybrid	Disposable	Hybrid	Disposable	Hybrid
Packaging (*n*)	4	6	0	2	3	5
Weight (kg)	0.07	0.26	0	0.2	0.04	0.2

^A^Including counting cards.

### Scope and system boundaries

**Figure 1:**
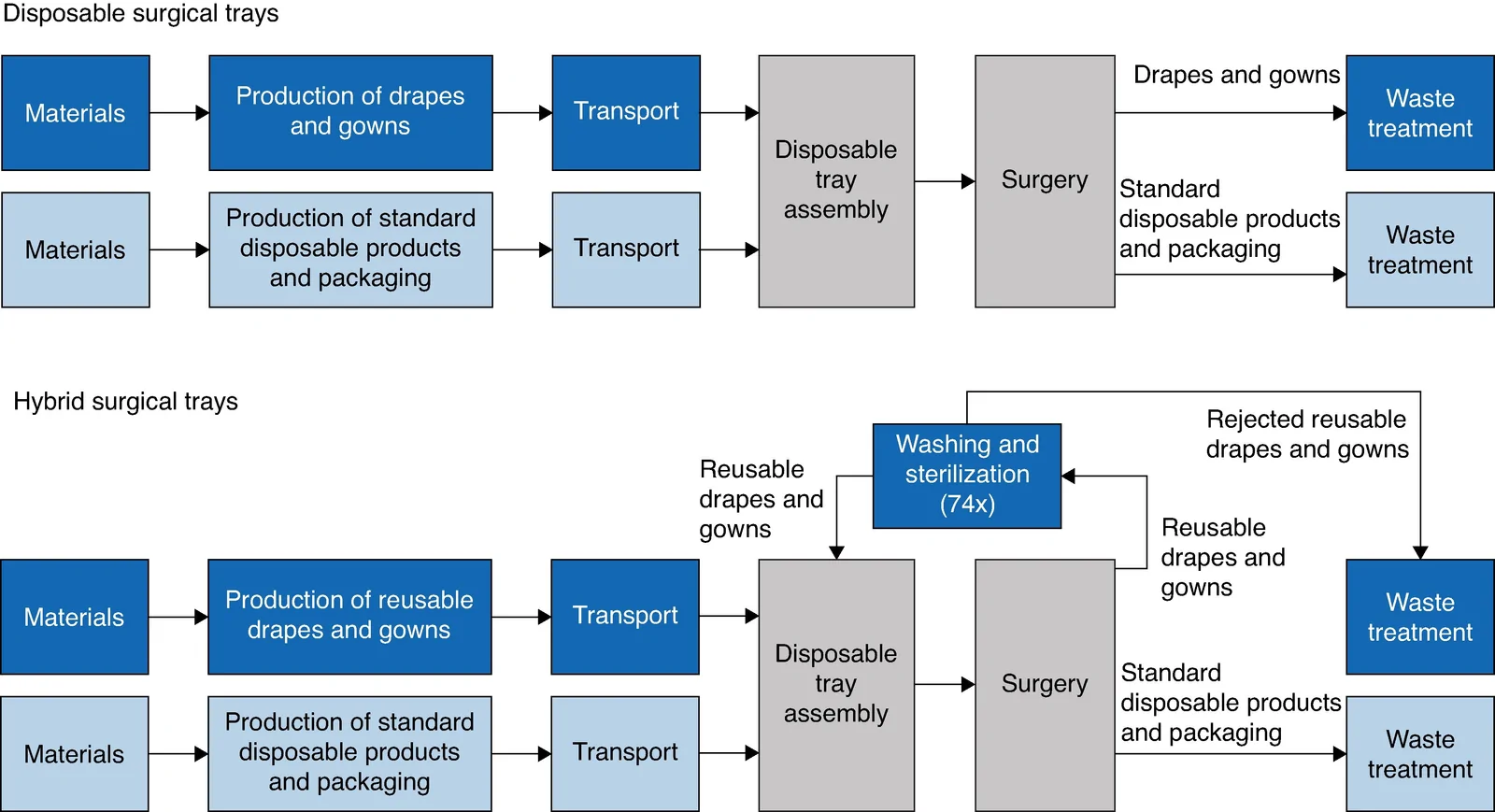
System boundaries of the model of disposable and hybrid surgical trays

The LCA focuses on the use of surgical trays in the Netherlands under current conditions. The system boundaries include all steps from the production of the products and packaging (for example, raw material extraction, conversion into disposable or reusable products, transportation), to washing and sterilization of reusable drapes and gowns (for hybrid trays only), and end-of-life incineration. These system boundaries are illustrated in the flow diagram in *Fig. 1*.

Furthermore, the following considerations are important for a full understanding of the system. First, the analysis focuses on the surgical tray only; environmental impacts from other aspects of surgery (for example, energy for equipment, anaesthesia, instrumental sets) are not included. Second, for the reusable drapes and gowns, a reuse rate of 74 times is assumed. According to the supplier, reusable products can be used up to 80 times. Approximately 15% of these products do not reach the maximum time, mainly due to loss. It is assumed that this 15% of reusable products is reused 40 times, on average. The average reuse rate of all reusable products is thereby estimated to be 74 times. Third, sterilization of the products after the production process is not included. Finally, at end-of-life, all products are incinerated without energy recovery, reflecting current practice for surgical waste in the Netherlands. In a sensitivity analysis, the effect of incineration with energy recovery is evaluated.

### Data sources and quality

Life cycle inventory data were collected to develop the rEUsable LCA model. First, the compositions of the products on the surgical trays were determined in order to assess the environmental impact of raw material extraction, production processes, and end-of-life incineration. Each product was individually weighed. Material compositions were identified using primary data from suppliers when available; otherwise, they were estimated based on supplier information for comparable products or through visual inspection. The material composition of the products in each tray is provided in *Supplementary methods S1*. Second, inventory data for washing and sterilization, such as energy and water usage, were collected based on primary data obtained from the external washing facility. Finally, transport distances were estimated based on the locations of production sites, the washing facility, and waste treatment facilities.

To assess the data quality of material composition, each approach (to determine material composition) is given a rating from 1 to 5 based on the pedigree matrix commonly used in LCA^13^. A rating of 1 represents the highest data quality, whereas a rating of 5 represents the most uncertain data. For all trays, more than 47% of materials (based on weight) are determined based on highest-quality data (rating 1). More details are provided in *Supplementary methods S2*.

### Modelling details

The foreground process data (for example, amount of electricity (kWh) used during washing) are combined with representative Ecoinvent (v3.11) background data sets (containing, for example, the carbon dioxide (CO_2_) and other emissions of average European electricity) to calculate environmental impacts. Background data sets are analysed in SimaPro software (SimaPro v10.2, PRé Sustainability B.V., Amersfoort, The Netherlands), using the ReCiPe 2016 Endpoint (H) V1.11 characterization method to generate results for 18 environmental impact indicators. These are further processed and combined in the Microsoft^®^ Excel-based LCA model containing the tray compositions.

Some products (for example, paper packaging) contain biogenic carbon. In this study a carbon-neutral approach has been used for biogenic carbon^14^. This means that biogenic CO_2_ capture and emissions (for example, from incineration at end-of-life) are not considered in the carbon footprint.

The full modelling details, including data sources and assumptions, are provided in *Supplementary methods S3*.

### Sensitivity analyses

Because certain modelling decisions are inevitably accompanied by uncertainty, sensitivity analyses were conducted to assess the robustness of the results. These analyses are introduced in *Table 2*, with full modelling details provided in *Supplementary methods S4*.

**Table 2:** Overview of sensitivity analyses

Sensitivity analysis	Rationale	Model changes
Reuse rate	The reuse rate of drapes and gowns in hybrid trays used in the baseline analysis (74 times) is based on estimations	Reuse rate is lowered to 50 times, 25 times, and 15 times
Geographical scope (with energy recovery)	In the baseline analysis, the geographical scope is the Netherlands (using the Dutch electricity mix and assuming incineration of discarded tray products without energy recovery). There are, however, substantial differences in the energy sources (and thus climate change impact) used in European countries. This difference in electricity mix has an even larger influence when assuming energy is recovered during incineration	Switched the electricity mix (for textile washing and sterilization and avoided electricity production due to energy recovery) to the average European, Swedish, or Polish mix, representing average, low-carbon, and high-carbon electricity mixes. Adapted end-of-life modelling for all disposable and hybrid trays to include energy recovery based on average regional MSWI net energy recovery efficiencies. Recovered electricity and heat are assumed to replace the conventional national electricity mix and natural gas-based heat, providing environmental impact credits (substitution approach)

MSWI, Municipal Solid Waste Incineration.

## Results

**Figure 2:**
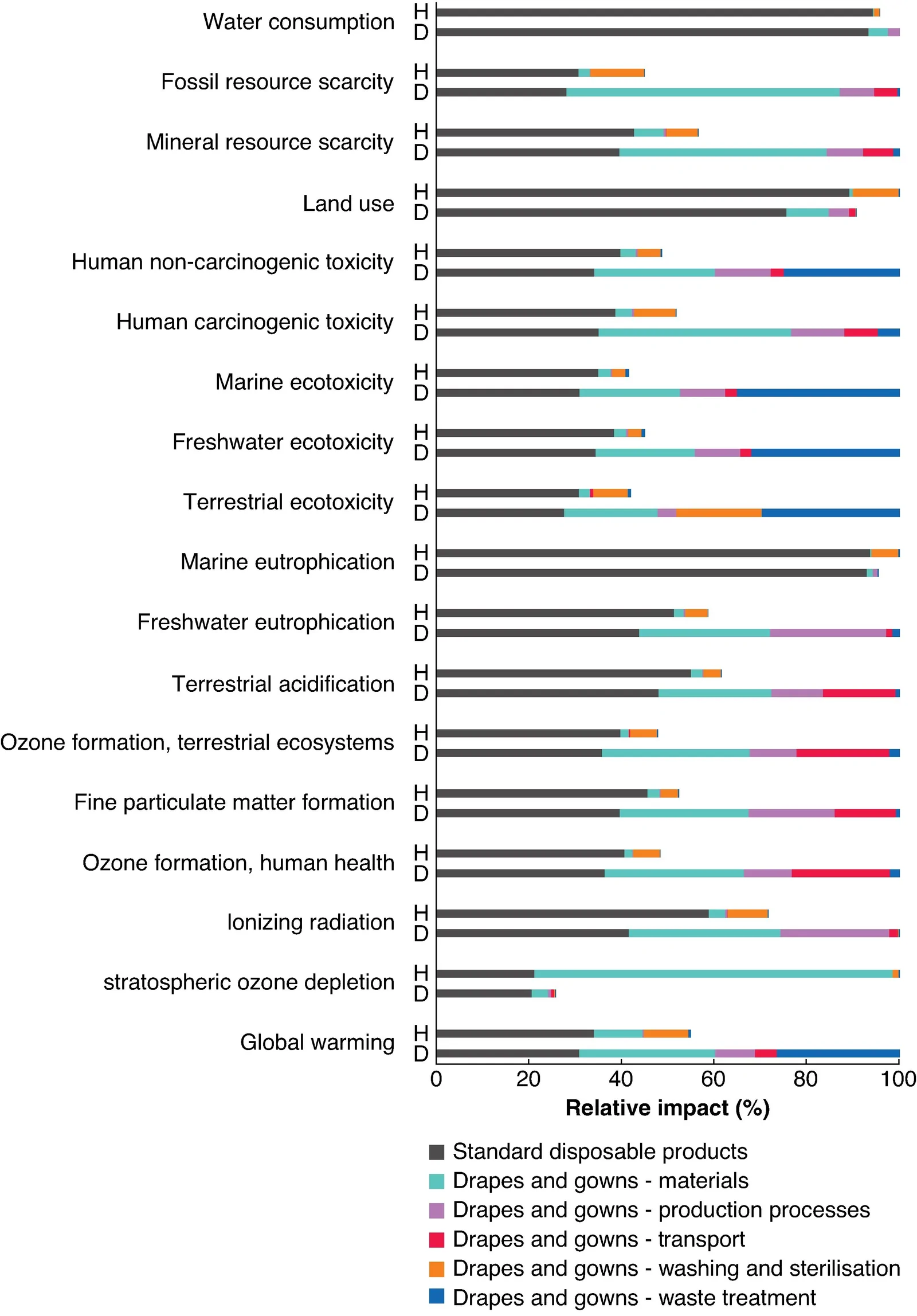
Relative impact on all environmental impact indicators of disposable and hybrid variants of average trays Note, the highest impact for each impact category is set to 100%. The graph shows a breakdown to life cycle phase for drapes and gowns; for (standard) disposable products, only the total impact is shown. D, disposable surgical trays; H, hybrid surgical trays.

The hybrid trays have the lowest environmental impact in almost all impact categories. In *Fig. 2*, the relative environmental impact results across all impact categories are illustrated for an average disposable and a hybrid tray. The results show that in 14 impact categories, the hybrid tray variant has a considerably lower impact (> 25%) than the disposable tray variant. In addition, in three impact categories (water consumption, land use and marine eutrophication), the impacts of both tray types are comparable (< 9% difference). In these categories, the environmental impact is largely (> 85%) determined by the production of cotton used in gauzes, which are present on both tray variants and categorized as standard disposable products (shown in grey in *Fig. 2*). In one impact category (stratospheric ozone depletion), the hybrid tray has a higher (286%) impact than the disposable tray. The high impact of the hybrid tray on stratospheric ozone depletion is caused by the expanded polytetrafluoroethylene (ePTFE) laminate in the drapes, contributing approximately 72% to the total impact.

**Figure 3:**
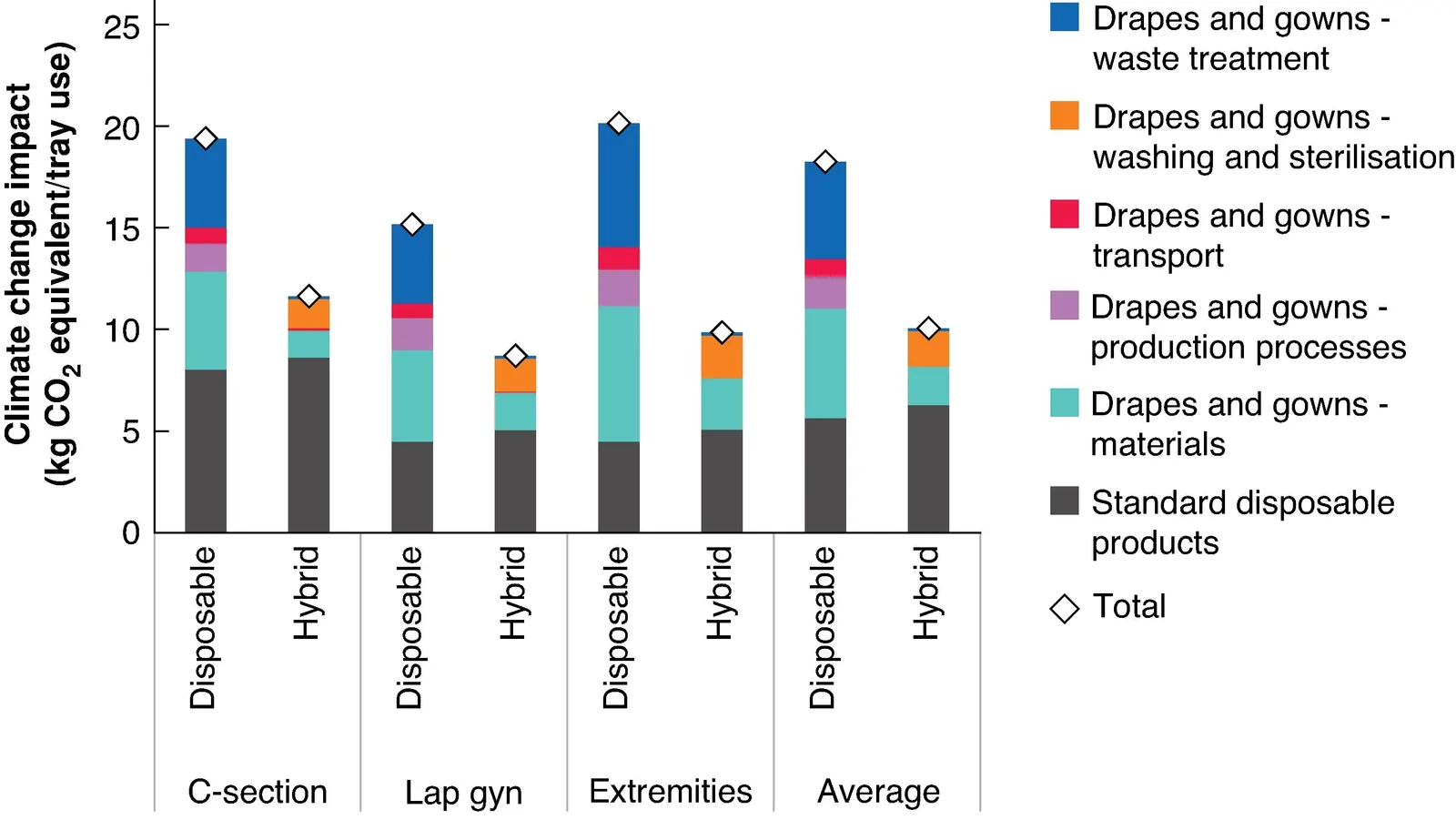
Climate change impact of the disposable and hybrid variants of three separate surgical trays, as well as the average impact of the three trays The graph shows a breakdown to life cycle phase for the drapes and gowns; for standard disposable products, only the total impact is shown. CO_2_, carbon dioxide; C-section, Caesarean section; Lap Gyn, gynaecological laparoscopy; Extremities, extremity surgery.

To examine the results on climate change in greater detail, *Fig. 3* illustrates the outcomes for the disposable and hybrid variants of the three tray types, as well as the average across all three. The impact of the standard disposable products (shown in grey in *Fig. 3*), which includes the production and end-of-life treatment of these items, is nearly identical between the disposable and hybrid trays. Minor differences are due to variations in packaging.

Overall, the use of a hybrid tray has a 40–51% lower climate change impact than the use of disposable trays across their entire life cycle. In disposable trays, the drapes and gowns contribute, on average, 69% to the total impact on climate change, representing the primary environmental hotspot. In the hybrid trays, this is reduced to 38% by using reusable drapes and gowns.

The differences in impact on climate change between the trays for C-sections, gynaecological laparoscopy, and extremity surgeries are mostly caused by the number of products on the trays and, to a lesser extent, by the composition.

The impacts of material extraction (show in light blue in *Fig. 3*), production (shown in yellow), transport (shown in green), and waste treatment (shown in purple) of drapes and gowns are considerably higher for the disposable than hybrid trays. This is due to the impacts of material extraction, production, transport, and waste treatment being distributed over their entire lifetime (74 uses), resulting in a relatively low impact per use.

The impact of material extraction (shown in light blue in *Fig. 3*) on the hybrid tray (88%) is determined by the use of ePTFE laminate in the reusable drapes. Production of the precursors of polytetrafluoroethylene (PTFE) emits strong greenhouse gases, resulting in a high estimated carbon footprint (63 kg CO_2_ equivalent/kg ePTFE laminate).

Furthermore, for the hybrid trays, the washing and sterilization processes (shown in orange in *Fig. 3*) account for approximately 45% of the total climate change impact.

In conclusion, the use of hybrid trays results in a lower environmental impact than the use of disposable trays. The impact on stratospheric ozone depletion would need to be considered extremely important compared with climate change and other impact categories to reach another conclusion. An analysis of the single score aggregating all midpoint impact indicators using the default ReCiPe 2016 weighting factors as implemented in SimaPro shows that the average hybrid tray has a 48% lower (that is, better) single score than the average disposable tray.

### Sensitivity analyses

The results of the sensitivity analyses show that lowering the reuse rate of drapes and gowns from 74 times to 50, 25, or 15 times increases the climate change impact of hybrid trays per surgery. The break-even point is 16 times; that is, if the reusable drapes and gowns are used less than 16 times, the climate change impact of disposable trays is lower than that of the hybrid variant. For most other environmental impact categories, the reuse break-even points are even lower, between three and 11 times (full details are provided in *Supplementary results S5*). Exceptions are land use and marine eutrophication, which have no break-even point, and stratospheric ozone depletion, which has a break-even point of 1727 uses. As noted previously, these impact categories are either those in which differences between hybrid and disposable trays are largely driven by the standard disposable products (land use, eutrophication) or in which the hybrid tray already exhibits a higher impact in the baseline scenario (ozone depletion).

This sensitivity analysis shows that, even at reuse rates lower than the 74 assumed in the baseline analysis, hybrid trays still have a lower environmental impact than disposable trays.

Changing the geographical scope, including different electricity mixes, only has a small effect on the climate change impact. The impacts of washing and sterilization are mostly caused by the combustion of natural gas, which is not influenced by the geographical scope. Adding energy recovery to the end-of-life incineration of products lowers the climate change impact of waste treatment. This effect is smaller for hybrid trays, because a smaller amount of material is sent to waste treatment per surgery (due to the 74-times reuse of drapes and gowns). As a result, the climate change reduction achieved by hybrid trays is reduced from 45% to 40% when energy recovery during waste treatment is included.

**Figure 4:**
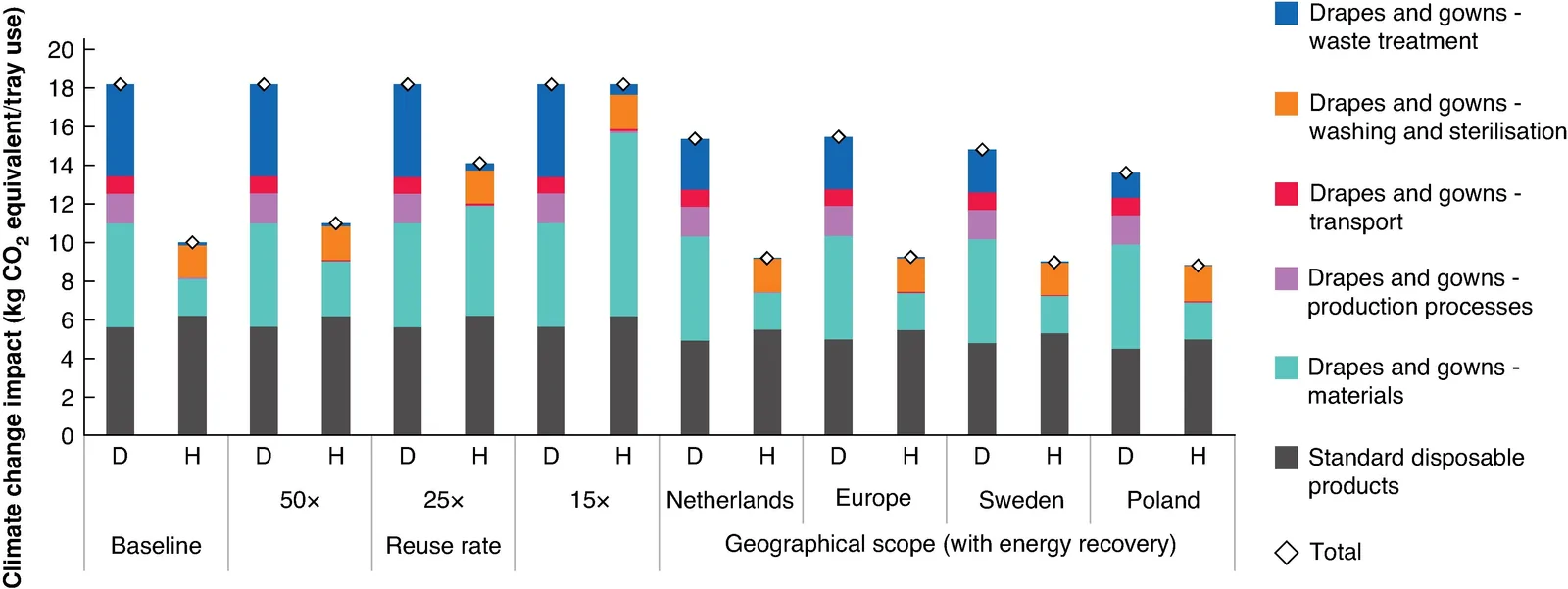
Sensitivity analyses of the climate change impacts for the mean disposable and hybrid trays CO_2_, carbon dioxide; D, disposable surgical trays; H, hybrid surgical trays.

*Figure 4* shows the results of the sensitivity analyses, illustrating how the climate change impact of the average disposable and hybrid trays varies across the different scenarios.

## Discussion

This study provides a comprehensive overview of the environmental impacts of widely used disposable procedure trays *versus* hybrid procedure trays. The results clearly show that replacing disposable drapes and gowns with reusables ones leads to a reduction in environmental impact in most impact categories, including a 40–51% reduction in climate change impact.

These findings are consistent with previous research on comparing disposable *versus* reusable alternatives in healthcare settings, such as a systematic review of LCAs on disposable *versus* reusable healthcare products by Keil *et al*.^15^, which showed that, based on 27 studies, most reusable alternatives perform better in most impact categories than their disposable alternatives.

While interpreting the results of this assessment, several uncertainties and limitations should be considered. The model incorporates a large number of products and materials (116 unique products, 21 materials), for which material composition, production processes, and transport had to be determined. This inevitably introduces various sources of uncertainty. For example, the material composition of some products had to be estimated based on visual inspection (see details in *Supplementary methods S3*). In addition, all materials are modelled using background data sets or literature data, which may not be fully representative of the production processes in practice. For example, plastic data sets do not account for the potential inclusion of additives, which can substantially influence environmental impacts. Transportation distances and modes also had to be estimated due to limited primary data and variations between hospitals. Finally, some processes, such as drape and gown assembly and tray sterilization, were not explicitly modelled due to data limitations.

However, it should be noted that these uncertainties affect both hybrid and disposable trays and are therefore unlikely to materially affect the comparison between the trays. In addition, the assumptions tend to be conservative for the hybrid trays. For example, any factors that underestimate the environmental impact of drape and gown production will have a greater effect on disposable trays because the impact of reusable drapes and gowns is divided over multiple uses.

Nevertheless, there are a few uncertainties for the reusable drapes and gowns that need to be highlighted. The ePTFE laminate used in reusable drapes and gowns is a key driver for its impacts on both climate change and ozone depletion^16^. Although the amount of ePTFE used is based on primary data, its production is modelled based on background data sets. Because this production is linked to highly potent greenhouse gases and ozone-depleting gases, small changes in these emissions can have a large effect on the climate change impact of reusable trays in general. Furthermore, the incineration of ePTFE is modelled as incineration of polyethylene terephthalate, which does not reflect the possible emission of per- and polyfluoroalkyl substances. However, this is not expected to have a large environmental impact, as it has been found that municipal incineration of PTFE is not a significant source of per- and polyfluoroalkyl substances^17^.

Finally, tray compositions vary widely in clinical practice. To account for this variability, three distinct tray types from different hospitals were included, enabling assessment of the environmental impacts associated with different drapes, gowns, and tray sizes. This approach enhances the generalizability of the present findings. Nevertheless, the exact reduction in impact varies depending on tray design.

The next phase of this research will focus on optimizing tray composition to include only clinically necessary items. A multicentre clinical trial is currently being conducted across three hospitals, where hybrid trays are used and evaluated during 450 procedures. Further reductions in environmental impact are expected through the elimination of unused products. The extent of this reduction can be estimated based on the environmental footprint per product determined in the present study. Findings from the ongoing trial will be presented in a subsequent publication. Additional strategies to minimize the environmental impact of surgical trays may involve the use of renewable energy sources for washing and sterilization, as well as reducing the footprint of individual products (for example, by developing lighter or smaller drapes and gowns or with less polluting materials). Moreover, to support the broader transition towards reusable textiles in healthcare, the economic and logistical implications of this shift will also be explored.

This study compared the environmental impacts of hybrid and disposable surgical trays using LCA in accordance with ISO 14040/44 standards. The findings show that hybrid trays consistently have lower environmental impacts across most impact categories, highlighting the environmental benefits of incorporating reusable materials in healthcare. These results provide an important evidence base to support a broader transition (back) to reusable drapes and gowns in the operating room.

## Supplementary Material

zrag139_Supplementary_Data

## Data Availability

The data supporting the findings of this study are available in the *Supplementary material*.
