# Supplementary material for: Environmental benefits of reintroducing reusable drapes and gowns in the operating room
Source: BJS Open. 2026 Sep 21;10(5):zrag139. doi: 10.1093/bjsopen/zrag139 (PMC13591279; doi:10.1093/bjsopen/zrag139)
Supplement: Supplementary file 1 [file zrag139_supplementary_data.docx]

**Environmental benefits of reintroducing reusable drapes and gowns in the Operating Room**

Florine I. de Haes^1,2^, Meis Uijttewaal^2^, Nicolaas H. Sperna Weiland^1,3^, Martijn L.M. Broeren^2^, Dorien A. Salentijn^1,2,4^

1: Centre for Sustainable Healthcare, Amsterdam UMC, Amsterdam, The Netherlands

2. Epidemiology and Data Science, Amsterdam UMC, Amsterdam, The Netherlands

2: CE Delft, Delft, The Netherlands

3: Department of Anesthesiology, Amsterdam UMC location University of Amsterdam, Amsterdam, The Netherlands

4: Department of Radiology and nuclear medicine, Amsterdam UMC location University of Amsterdam, Amsterdam, The Netherlands

**Corresponding author:**

Dorien Salentijn, Meibergdreef 9 1105 AZ Amsterdam

[d.a.salentijn@amsterdamumc.nl](mailto:d.a.salentijn@amsterdamumc.nl)

**Supplementary Materials - Index**

| **Supplementary Methods** |  |
| --- | --- |
| S.I. 1: Product composition | *page 1* |
| S.I. 2: Data quality product material composition | *page 14* |
| S.I. 3: Life cycle inventory (LCI) and modelling details | *page 15* |
| S.I. 4: Modelling details sensitivity analyses | *page 23* |
| **Supplementary Results** |  |
| S.I. 5: Results break-even analysis reuse rate | *page 25* |
| **References** | *page 26* |

**Supplementary Methods**

**S.I. 1: Product composition**

Six trays, two of each type, were provided by the rEUsable project for the LCA. Each product on both the disposable and hybrid tray was weighed and its composition was determined. Where possible the different materials/components of the product were weighted separately, after disassembling the products. Tables SI1 – SI9 present the items included on the trays along with their material composition and weight.

The material composition was determined using different approaches, with the following hierarchy:

1. The material composition was stated on the product
2. The material composition for the product on the tray was provided by the supplier
3. The material composition of a comparable product was provided by the supplier
4. The material composition was estimated

**Table SI1 - C-section tray – Standard disposable products**

| Product | # on tray | Total weight | PE^1^ | PP | Flexible^2^ | Velcro | Metal^3^ | Cotton | PP (textile) | Paper | Other materials^4^ | Reliability |
| --- | --- | --- | --- | --- | --- | --- | --- | --- | --- | --- | --- | --- |
|  | Amount | g/piece | g | g | g | g | g | g | g | g | g |  |
| Suction tube yankauer 24Ch 25cm round tip, 4 eyes with handle | 1 | 17.1 |  | 17.1 |  |  |  |  |  |  |  | 2 |
| Suction tube / Aspiration tube OD 30Ch 3,5m, F/F | 1 | 199.5 |  |  | 199.5^a^ |  |  |  |  |  |  | 1 |
| Foley catheter, 14Ch, 2-way silicone, balloon 5-10ml, 40cm | 1 | 10.1 |  |  | 10.1^b^ |  |  |  |  |  |  | 4 |
| Maternity pad nonwoven 11x35cm | 1 | 16.8 |  |  |  |  |  | 15.1 | 1.7 |  |  | 3 |
| Gauze swab round nr.5 X-ray detectable (XRD), white | 2 | 21.7 |  |  |  |  |  | 21.2 |  |  | 0.5^c^ | 1 |
| Count card, gauze swab nr.5 XRD | 1 | 0.9 |  |  |  |  |  |  |  |  | 0.9^d^ | 3 |
| Abdominal compress/ gauze 45x70cm 3p XRD white , bundled per 5pcs | 2 | 142.8 |  |  |  |  |  | 138.1 | 3.8 |  | 0.9^d^ | 1 |
| Count card gauze swab nr.5 XRD | 1 | 0.9 |  |  |  |  |  |  |  |  | 0.9^d^ | 3 |
| Scalpel blade 10 cs | 1 | 1.1 |  |  |  |  | 0.6^a^  0.5^b^ |  |  |  |  | 3 |
| Diathermy electrode knife 7,0cm stainless steel | 1 | 2.2 |  | 0.2 |  |  | 2.0^a^ |  |  |  |  | 3 |
| Scalpel blade 20 cs | 1 | 1.4 |  |  |  |  | 0.7^a^  0.7^b^ |  |  |  |  | 3 |
| Bowl 1000ml blue graduated | 1 | 29.5 |  | 29.5 |  |  |  |  |  |  |  | 1 |
| Bowl 500ml transparent graduated | 1 | 17.0 |  | 17.0 |  |  |  |  |  |  |  | 1 |
| Bowl 250ml blue graduated | 1 | 9.1 |  | 9.1 |  |  |  |  |  |  |  | 1 |
| Bowl 60ml transparent | 1 | 3.7 |  | 3.7 |  |  |  |  |  |  |  | 1 |
| Self-adhesive diathermy pouch 40x35cm | 1 | 21.4 |  |  | 19.4^c^ |  |  |  |  | 2.0 |  | 1 |
| Urinebag 2000ml, tube 90cm, with valve | 1 | 41.3 | 5.8^a^ |  | 35.5^a^ |  |  |  |  |  |  | 1 |
| Collection pouch | 1 | 148.6 |  |  | 148.6^c^ |  |  |  |  |  |  | 3 |
| Green reinforced lamp handle cover | 1 | 4.2 |  |  | 4.2^a^ |  |  |  |  |  |  | 5 |
| Umbilical cord clamp, white | 3 | 12.6 |  | 12.6 |  |  |  |  |  |  |  | 5 |
| Lamp handle cover | 1 | 12.5 |  | **4.1** | 8.4^a^ |  |  |  |  |  |  | 5 |
| Syringe 10ml, Luer slip, 3-part, centric tip | 1 | 7.5 |  | 6.4 |  |  |  |  |  |  | 1.1^b^ | 5 |
| See-through sheet | 1 | 99.4 |  |  |  |  |  |  |  |  |  | 3 |
| Crepe paper 60x60cm 60g/m² white | 1 | 20.9 |  |  |  |  |  |  |  | 20.9 |  | 3 |
| Paper bag 19x25x6,5cm white | 1 | 9.2 |  |  |  |  |  |  |  | 9.2 |  | 3 |
| Label 1x4cm white Arterial  Label 1x4cm white Venous  Label 1x4cm white SE NaCl  Label 1,6x5cm red H2O | 1 | 0.5 |  |  |  |  |  |  |  |  | 0.5^d^ | 3 |
| Crepe paper 75x75cm 60g/m² green | 1 | 32.7 |  |  |  |  |  |  |  | 32.7 |  | 3 |
| Paper bag 11x19x4,5cm wit | 1 | 4.8 |  |  |  |  |  |  |  | 4.8 |  | 3 |

1: a=HDPE, b=LDPE

2: a=PVC, b=silicone, c=PEVA

3: a=iron, b=aluminium

4: a=glue, b=rubber, c=elastic, d=sticker

**Table SI2 – Lap gyn tray – Standard disposable products**

| Product | # on tray | Total weight | PE^1^ | PP | Flexible^2^ | Velcro | Metal^3^ | Cotton | PP (textile) | Paper | Other materials^4^ | Reliability |
| --- | --- | --- | --- | --- | --- | --- | --- | --- | --- | --- | --- | --- |
|  | Amount | g/piece | g | g | g | g | g | g | g | g | g |  |
| Insufflation tube, 300 cm | 1 | 144.9 | 2.5^b^ | 12.8 | 129.6^a^ |  |  |  |  |  |  | 4 |
| Desufflation tube ,425 cm | 1 | 191.0 | 2.5^b^ | 12.8 | 175.7^a^ |  |  |  |  |  |  | 4 |
| Scalpel blade, no. 15, carbon steel | 1 | 0.8 |  |  |  |  | 0.3^a^  0.5^b^ |  |  |  |  | 3 |
| Bowl, 250 ml | 2 | 10.8 |  | 10.8 |  |  |  |  |  |  |  | 1 |
| Kidney dish,700ML | 1 | 28.8 |  | 28.8 |  |  |  |  |  |  |  | 1 |
| Diathermy pouch, 46x24 cm, 3 compoartments | 2 | 32.3 |  |  | 27.5^c^ |  |  |  |  |  | 4.8^d^ | 1 |
| Diathermy pouch (small) | 1 | 44.9 |  |  | 44.9^c^ |  |  |  |  |  |  | 1 |
| Self-adhesive fluid collection pouch (50 x 50 cm) with suction port | 1 | **73.9** | 2.9^a^ |  | 71.0^c^ |  |  |  |  |  |  | 1 |
| Needle counter, foam/magentic, red | 1 | 50.3 |  | 45.4 | 4.9^a^ |  |  |  |  |  |  | 5 |
| Cable holder, 30 x 2.5 cm, velcro | 1 | 5.2 |  |  |  | 5.2 |  |  |  |  |  | 1 |
| Gauze swab, 10 x 10 cm, XR, 12-ply | 1 | 31.4 |  |  |  |  |  | 31.4 |  |  |  | 1 |
| Count strip, gauze, 10 x 10 cm, 10 pcs, XRD | 1 | 1.1 |  |  |  |  |  |  |  |  | 1.1^d^ | 3 |
| Lamp cover, purple | 1 | 5.0 |  |  | 5.0^a^ |  |  |  |  |  |  | 5 |

1: a=HDPE, b=LDPE

2: a=PVC, b=silicone, c=PEVA

3: a=iron, b=aluminium

4: a=glue, b=rubber, c=elastic, d=sticker

**Table SI3 – Extremities tray – Standard disposable products**

| Product | # on tray | Total weight | PE^1^ | PP | Flexible^2^ | Velcro | Metal^3^ | Cotton | PP (textile) | Paper | Other materials^4^ | Reliability |
| --- | --- | --- | --- | --- | --- | --- | --- | --- | --- | --- | --- | --- |
|  | Amount | g/piece | g | g | g | g | g | g | g | g | g |  |
| Yankauer suction tube, 18Ch, 27cm, 6 holes, round tip | 1 | 13.3 |  | 13.3 |  |  |  |  |  |  |  | 2 |
| Suction hose / aspiration tube OD 30Ch, 4m, f/f | 1 | 199.5 |  |  | 199.5^a^ |  |  |  |  |  |  | 1 |
| Gauze compress 10x20cm, 17 threads, 12-ply, XRD, white, 5 pcs | 2 | 58.8 |  |  |  |  |  | 58.8 |  |  |  | 1 |
| Count card NL 10, gauze/compress 10x20cm XRD | 2 | 1.0 |  |  |  |  |  |  |  |  | 1.0^d^ | 2 |
| Scalpel blade no. 21, steril | 1 | 1.5 |  |  |  |  | 0.9^a^  0.6^b^ |  |  |  |  | 3 |
| Scalpel blade no.10, sterile | 1 | 1.1 |  |  |  |  | 0.6^a^  0.5^b^ |  |  |  |  | 3 |
| Scalpel blade no. 15, sterile | 1 | 0.8 |  |  |  |  | 0.3^a^  0.5^b^ |  |  |  |  | 3 |
| Tray/plateau 25x14x5cm 1575ml blue | 3 | 36.8 |  | 36.8 |  |  |  |  |  |  |  | 3 |
| Bowl 500ml transparent | 2 | 17.0 |  | 17.0 |  |  |  |  |  |  |  | 1 |
| Bowl 250ml blue | 1 | 8.8 |  | 8.8 |  |  |  |  |  |  |  | 1 |
| Bowl 250ml transparent | 1 | 9.1 |  | 9.1 |  |  |  |  |  |  |  | 1 |
| Self-adhesive fluid collection pouch 40x35cm | 1 | 20.1 |  |  | 18.2^c^ |  |  |  |  | 1.9 |  | 1 |
| Self-adhesive cable holder 2,5x30cmvelcro | 1 | 5.2 |  |  |  | 5.2 |  |  |  |  |  | 1 |
| Label sheet A5, 20self-adhesive labels 1x4cm | 1 | 12.6 | 4.4^b^ |  |  |  |  |  |  | 5.1 | 3.1^d^ | 3 |
| Surgical-tape 9x55cm transparent | 2 | 8.3 | 2.2^b^ |  |  |  |  |  |  | 4.6 | 1.5^a^ | 3 |
| Surgical-tape 9x49cm | 1 | 8.4 |  |  |  |  |  |  | 3.0 | 3.4 | 2.0^a^ | 1 |
| Syringe 60ml Luer lip,3d-part, catheter tip, centric | 1 | 34.6 |  | 30.7 |  |  |  |  |  |  | 3.9^b^ | 1 |
| Paper bag 18x38x9,5cm white | 1 | 7.9 |  |  |  |  |  |  |  | 7.9 |  | 3 |
| Paper bag 14x25x7,5cm white | 1 | 6.2 |  |  |  |  |  |  |  | 6.2 |  | 3 |

1: a=HDPE, b=LDPE

2: a=PVC, b=silicone, c=PEVA

3: a=iron, b=aluminium

4: a=glue, b=rubber, c=elastic, d=sticker

**Table SI4 – C-section tray: Drapes and gowns disposable tray**

| Product | # on tray | Total weight | LDPE | PP | PVC | Velcro | PET (textile) | Cotton | PP (textile)^1^ | Paper | Other materials^2^ | Reliability |
| --- | --- | --- | --- | --- | --- | --- | --- | --- | --- | --- | --- | --- |
|  | Amount | g/piece | g | g | g | g | g | g | g | g | g |  |
| Cesarean section drap, self-adhesive, 175/250x300cm, film window 32x38cm | 1 | 498.6 | 139.0 | 3.6 | 7.2 |  |  |  | 277.9^a^ | 24.6 | 46.3^a^ | 3 |
| Baby drape 90x120cm white | 2 | 72.0 |  |  |  |  |  |  | 72.0^a^ |  |  | 5 |
| Cover drape 150x175cm | 1 | 195.2 | 58.6 |  |  |  |  |  | 117.1^a^ |  | 19.5^a^ | 3 |
| Mayo stand cover 79x145cm, reinforced | 1 | 306.0 | 260.1 |  |  |  |  |  | 30.6^a^ |  | 15.3^a^ | 1 |
| Surgical gown, sterile, size XL-L | 2 | 137.1 |  |  |  | 1.4 | 8.3 |  | 124.7^b^ | 2.6 | 0.1^e^ | 1 |
| Instrument table cover, 150x240cm, reinforced | 1 | 181.0 | 132.1 |  |  |  |  |  | 38^a^ |  | 10.9 | 1 |

1: a=non-woven, b=SMS

2: a=glue, b=rubber, c=elastic, d=sticker, e=finish

**Table SI5 – Lap gyn tray: Drapes and gowns disposable tray**

| Product | # on tray | Total weight | LDPE | PP | PVC | Velcro | PET (textile) | Cotton | PP (textile)^1^ | Paper | Other materials^2^ | Reliability |
| --- | --- | --- | --- | --- | --- | --- | --- | --- | --- | --- | --- | --- |
|  | Amount | g/piece | g | g | g | g | g | g | g | g | g |  |
| Gynaecological drape, 2-3 ply, 280x180cm | 1 | 689.3 | 197.3 |  |  |  |  |  | 394.6^a^ | 31.6 | 65.8^a^ | 3 |
| Instrument table cover, 195x150cm | 1 | 191.0 | 139.4 |  |  |  |  |  | 40.1^a^ |  | 11.5^a^ | 1 |
| Mayo stand cover, 76x145cm, medium | 1 | 127.5 | 108.4 |  |  |  |  |  | 12.8^a^ |  | 6.4^a^ | 1 |
| Surgical gown, size L-XL | 1 | 121.9 |  |  |  | 1.1 | 6.1 |  | 112.3^b^ | 2.3 | 0.1^e^ | 1 |
| Surgical gown, size XL-XL | 2 | 147.8 |  |  |  | 2.4 | 5.8 |  | 137.4^b^ | 2.1 | 0.1^e^ | 1 |
| Drape with adhesive tape, 75x75cm, 2-ply | 1 | 37.2 | 9.9 |  |  |  |  |  | 19.8^a^ | 4.2 | 3.3^a^ | 3 |

1: a=non-woven, b=SMS

2: a=glue, b=rubber, c=elastic, d=sticker, e=finish

**Table SI6 –Extremities tray: Drapes and gowns disposable tray**

| Product | # on tray | Total weight | LDPE | PP | PVC | Velcro | PET (textile) | Cotton | PP (textile)^1^ | Paper | Other materials^2^ | Reliability |
| --- | --- | --- | --- | --- | --- | --- | --- | --- | --- | --- | --- | --- |
|  | Amount | g/piece | g | g | g | g | g | g | g | g | g |  |
| Self-adhesive cover drape, 300x180cm, reinforced | 1 | 320.9 | 94.3 |  |  |  |  |  | 188.6^a^ | 6.6 | 31.4^a^ | 3 |
| Self-adhesive cover drape, 180x180cm, reinforced | 1 | 195.8 | 57.1 |  |  |  |  |  | 114.1^a^ | 5.6 | 19.0^a^ | 1 |
| Self-adhesive split drape, 225x260cm, split 20x102cm | 1 | 406.8 | 118.6 |  |  |  |  |  | 237.2^a^ | 11.4 | 39.5^a^ | 3 |
| Instrument table cover, reinforced, 150x190cm, absorbent, | 1 | 261.3 | 210.1 |  |  |  |  |  | 51.2^a^ |  |  | 1 |
| Self-adhesive cover drape, 120x100cm, reinforced | 1 | 91.1 | 25.26 |  |  |  |  |  | 50.52^b^ | 6.9 | 8.42^a^ | 3 |
| Mayo stand cover, 79x145 cm, reinforced | 1 | 306.0 | 260.1 |  |  |  |  |  | 30.6^a^ |  | 15.3^a^ | 1 |
| Surgical gown, size XL-L | 2 | 137.1 |  |  |  | 1.4 | 8.3 |  | 124.7^b^ | 2.6 | 0.1^e^ | 1 |
| Surgical gown, size L-L | 1 | 100.5 |  |  |  | 1.3 | 6.6 |  | 89.9^b^ | 2.6 | 0.1^e^ | 1 |
| Instrument table cover, 150x190cm, reinforced, | 1 | 238.7 | 174.3 |  |  |  |  |  | 50.1^a^ |  | 14.3^a^ | 1 |

1: a=non-woven, b=SMS

2: a=glue, b=rubber, c=elastic, d=sticker, e=finish

**Table SI7 – C-section tray: Reusable drapes and gowns hybrid tray**

| Product | # on tray | Total weight | LDPE | PET microfiber | ePTFE laminate | Paper | Other materials^1^ | Adhesive layer^2^ | Reliability |
| --- | --- | --- | --- | --- | --- | --- | --- | --- | --- |
|  | Amount | g/piece | g | g | g | g | g | m |  |
| Cesarean section drape | 1 | 1400.0 |  |  | 1400.0 |  |  | 1.8^a^ | 1 |
| Baby drape | 2 | 180.4 |  | 175.0 |  |  | 5.4^e^ | 0.7^a^ | 1 |
| Cover drape | 1 | 368.3 |  | 354.0 |  |  | 14.3^e^ |  | 1 |
| Mayo stand cover | 1 | 325.8 |  | 320.0 |  |  | 5.8^e^ |  | 1 |
| Surgical gown | 2 | 515.0 |  | 500.0 |  |  | 15.0^e^ |  | 1 |
| Instrument table cover, 150x175cm | 1 | 367.1 |  | 354.0 |  |  | 13.1^e^ |  | 1 |
| Laundry bag | 1 | 18.0 | 18.0 |  |  |  |  |  | 3 |
| Packaging | 1 | 176.5 |  |  |  | 176.5 |  |  | 3 |

1: a=glue, b=rubber, c=elastic, d=sticker, e=finish

2: a=5cm width, b=10cm width

**Table SI8 – Lap gyn tray: Reusable drapes and gowns hybrid tray**

| Product | # on tray | Total weight | LDPE | PET microfiber | ePTFE laminate | Paper | Other materials^1^ | Adhesive layer^2^ | Reliability |
| --- | --- | --- | --- | --- | --- | --- | --- | --- | --- |
|  | Amount | g/piece | g | g | g | g | g | m |  |
| Drape, 280x180cm | 1 | 1800.0 |  |  | 1800.0 |  |  | 1.8^a^  0.4^b^ | 1 |
| Instrument table cover (195x175) | 1 | 371.1 |  | 354.0 |  |  | 17.1^e^ |  | 1 |
| Mayo stand cover | 1 | 325.8 |  | 320.0 |  |  | 5.8^e^ |  | 1 |
| Surgical gown, size L-XL | 2 | 515.0 |  | 500.0 |  |  | 15.0^e^ |  | 1 |
| Surgical gown, size XL-XL | 1 | 590.0 |  | 575.0 |  |  | 15.0^e^ |  | 1 |
| Drape (75x75cm) | 1 | 139.0 |  |  | 139.0 |  |  | 0.8^a^ | 1 |
| Laundry bag | 1 | 18.0 | 18.0 |  |  |  |  |  | 3 |
| Packaging | 1 | 176.5 |  |  |  | 176.5 |  |  | 3 |

1: a=glue, b=rubber, c=elastic, d=sticker, e=finish

2: a=5cm width, b=10cm width

**Table SI9 – Extremities tray: reusable drapes and gowns hybrid tray**

| Product | # on tray | Total weight | LDPE | PET microfiber | ePTFE laminate | Paper | Other materials^1^ | Adhesive layer^2^ | Reliability |
| --- | --- | --- | --- | --- | --- | --- | --- | --- | --- |
|  | Amount | g/piece | g | g | g | g | g | m |  |
| Cover drape 300x180 | 1 | 752.0 |  |  | 752.0 |  |  | 0.9^a^ | 1 |
| Cover drape 180x180 | 1 | 721.0 |  |  | 721.0 |  |  | 1.0^a^ | 1 |
| Split drape 225x260 | 1 | 1200.0 |  |  | 1200.0 |  |  | 1.5^a^  0.2^b^ | 1 |
| Instrument table cover 150x190 | 1 | 368.3 |  | 354.0 |  |  | 14.3^e^ |  | 1 |
| Cover drape 120x100 | 1 | 85.0 |  | 85.0 |  |  |  |  | 1 |
| Mayo stand cover 79x145 | 1 | 325.7 |  | 320 |  |  | 5.7^e^ |  | 1 |
| Surgical gown | 3 | 515.0 |  | 500.0 |  |  | 15.0^e^ |  | 1 |
| Instrument table cover 150x190 | 1 | 368.3 |  | 354.0 |  |  | 14.3^e^ |  | 1 |
| Laundry bag | 1 | 18.0 | 18.0 |  |  |  |  |  | 3 |
| Packaging | 1 | 177.3 |  |  |  | 177.3 |  |  | 3 |

1: a=glue, b=rubber, c=elastic, d=sticker, e=finish

2: a=5cm width, b=10cm width

**S.I. 2: Data quality product material composition**

The composition (weight, materials) of the products used in the procedure trays was determined using different approaches. The reliability of the material composition used has been categorized using the reliability indicator from the Pedigree Matrix frequently applied in LCA studies^1^. The Pedigree Matrix distinguishes five categories for data reliability, with a score of 1 representing the highest reliability and 5 the lowest:

1. Verified data based on measurements: in this study it concerns products for which composition data was provided by primary sources.
2. Verified data partly based on assumptions or non-verified data based on measurements: products for which composition data was provided by Evercare Medical, but no dataset for the material was available in the background databases, so a proxy dataset has been used.
3. Nonverified data partly based on assumptions: either products for which the material composition was estimated by visual inspection, with great certainty (e.g. paper packaging or PP bowls with the material composition printed on the outside) or products for which the same composition was assumed as comparable products for which Evercare Medical provided information (mostly disposable drapes and gowns).
4. Qualified estimate: products for which the material composition was estimated based on visual inspection, but which likely contain additives which are not included.
5. Nonqualified estimate or unknown origin: products for which the material composition was estimated by visual inspection, but with great uncertainty.

Table SI10 shows an overview of the distribution of product weight over the reliability categories for each tray.

**Table SI10: The distribution of products (based on weight) over the reliability categories for each tray.**

|  | **C-section** | | **Lap gyn** | | **Extremities** | |
| --- | --- | --- | --- | --- | --- | --- |
|  | Disposable | Hybrid | Disposable | Hybrid | Disposable | Hybrid |
| 1 | 53% | 88% | 47% | 89% | 64% | 94% |
| 2 | 1% | 0% | 0% | 0% | 0% | 0% |
| 3 | 39% | 10% | 34% | 4% | 35% | 6% |
| 4 | 0% | 0% | 16% | 7% | 0% | 0% |
| 5 | 8% | 1% | 3% | 1% | 0% | 0% |

**S.I. 3: Life cycle inventory (LCI) and modelling details**

**LCI datasets**

The environmental impact of the materials was determined using ecoinvent datasets. Table SI11 shows the datasets selected for each material. For the following materials a proxy dataset was selected: elastic (rubber as proxy), stickers (LDPE as proxy), ePTFE membrane (tetrafluorethylene as proxy).

**Table SI11 - Modelling details materials**

| **Material** | **Ecoinvent dataset** |
| --- | --- |
| HDPE | Polyethylene, high density, granulate {GLO}\| market for |
| LDPE | Polyethylene, linear low density, granulate {GLO}\| market for |
| PVC | Polyvinyl chloride, unspecified polymerisation, weighted average \| market for |
| PP | Polypropylene, granulate {GLO}\| market for |
| PET | Polyethylene terephthalate, granulate, amorphous {GLO}\| market for |
| ePTFE laminate | 44% Tetrafluoroethylene {GLO}\| market for  56% Polyethylene terephthalate, granulate, amorphous {GLO}\| market for  56% Weaving, synthetic fibre {GLO}\| market for |
| Polyester microfiber | 99% Polyethylene terephthalate, granulate, amorphous {GLO}\| market for  1% Carbon fibre reinforced plastic, injection moulded {GLO}\| market for |
| Velcro | 80% Nylon 6-6 {RER}\| market for  20% Polyethylene terephthalate, granulate, amorphous {GLO}\| market for |
| Silicone | Silicone product {RER}\| market for |
| Iron | Steel, low-alloyed {GLO}\| market for |
| Aluminium | Aluminium, primary, ingot {IAI Area, EU27 & EFTA}\| market for |
| Cotton | Yarn, cotton {GLO}\| market for |
| Paper | Tissue paper {RER}\| tissue paper production, virgin |
| Rubber | Synthetic rubber {GLO}\| market for |
| Elastic | Modelled as rubber |
| Sticker | Modelled as LDPE |
| PEVA | Ethylene vinyl acetate copolymer {RoW} \| market for |
| Glue | Acrylic binder, with water, in 54% solution state {RoW} \| market for |
| Finish | See information below |

On several drapes and gowns a water-repellent finish is used. According to the supplier of the rEUsable drapes and gowns C6 fluorocarbon finish is used. This finish is also assumed to be used for the disposable drapes and gowns. The model of the production of C6 fluorocarbon finish is based on the MIDWOR study^2^. The amount of finish for the reusable drapes and gowns is based on the assumption that 5 grammes of finish per m^2^ of textile is used. The surface area of the drapes and gowns was estimated. For the disposable drapes and gowns the amount of finish used was based on information provided by the supplier. Tables SI12 – SI3 provide the modelling details of the finish.

**Table SI12 - Inventory data for the production of 1 kg of finish product**

| **Input** | **Unit** | **Amount** | **Details** |
| --- | --- | --- | --- |
| Fluorinated acrylic copolymer | kg | 0.6 | See Table SI13 |
| Citric acid | kg | 0.06 | *Citric acid {GLO}\| market for* |
| Ethylene glycol | kg | 0.3 | *Ethylene glycol {RoW} \| market for* |
| Electricity | kWh | 0.003 | *Electricity, high voltage {CN}\| market group for* |
| Natural gas | Nm3 | 0.006 | *Heat, district or industrial, natural gas {RoW} \| heat production, natural gas, at industrial furnace >100kW* |

**Table SI13 - Inventory data for the production of 1 kg of fluorinated acrylic copolymer**

| **Input** | **Unit** | **Amount** | **Details** |
| --- | --- | --- | --- |
| Tetrafluoroethylene | kg | 1.07 | The production process has a yield of 80%. The polymer consists for 85.8% of tetrafluoroethylene.  *Tetrafluoroethylene {GLO}\| market for* |
| Iodine | kg | 0.10 | The other main constituent is iodine pentafluoride. It is assumed that this makes up the other 14.2%.  *Iodine {GLO}\| market for* |
| Fluorine | kg | 0.08 | The other main constituent is iodine pentafluoride. It is assumed that this makes up the other 14.2%.  *Fluorine, liquid {RoW} \| market for* |
| Electricity | kWh | 0.0015 | *Electricity, high voltage {CN}\| market group for* |
| Natural gas | Nm3 | 0.003 | *Heat, district or industrial, natural gas {RoW} \| heat production, natural gas, at industrial furnace >100kW* |

**Production processes**

The production process is based on the material of which the product is made. Most materials are coupled with one production process. For some materials two production processes are listed (e.g. PP textile can be either produced as non-woven textile or as SMS cloth), and the choice is made depending on the type of product. For a few materials, the production process is already included in the model or in the ecoinvent dataset of the material production.

Only the production of basic products from raw materials (e.g. extrusion or blow moulding of plastic granulate, weaving of textile fibers) is included in the model. Assembly of different components of products is not included. Sterilisation of the products after the production process and packaging of the products (other than the packaging in which they are on tray) is also not included. Table SI14 shows the modelling details of the production processes.

**Table SI14 – Modelling details for production processes linked to type of material**

| **Type of material** | **Production process** | **Ecoinvent dataset** |
| --- | --- | --- |
| LDPE, PVC, Velcro, PEVA, Silicone | Extrusion | Extrusion, plastic film {GLO}\| market for |
| HDPE, PP | Blow moulding | Injection moulding {GLO}\| market for |
| Iron | Tempering | Impact extrusion of steel, hot, tempering {GLO}\| market for |
| Aluminium | Sheet rolling | Sheet rolling, steel {GLO}\| market for |
| Velcro, polyester textile, cotton, polyester microfiber | Weaving | Weaving, synthetic fibre {GLO} |
| PP textile | Non-woven textile | Textile, nonwoven polypropylene {RoW} \| textile production (Adjustments: PP input set to zero) |
| PP textile | SMS | textile, non-woven polypropylene {IN}\| textile production, non-woven polypropylene, spun bond (Adjustments: PP input set to zero, energy input multiplied by two to account for multiple layers) |
| ePTFE laminate, paper, glue, rubber, elastic, sticker, finish | Production process not modelled separately (already included in material production, see Table SI1) | n.a. |

**Transport**

All disposable products are assumed to be produced in China and transported by boat (20,000 km) and truck (1,500 km) to the port of Rotterdam. The reusable drapes and gowns are produced in Poland. They are assumed to be transported to the Netherlands by truck (1,200 km). Details are provided in Table SI15.

**Table SI15 – Inventory data for transport mode for disposable and reusable products**

| **Transport mode** | **China** | **Europe** | **Ecoinvent dataset** |
| --- | --- | --- | --- |
| Transport by truck | 1,500 km | 1,200 km | Transport, freight, lorry 16-32 metric ton, EURO6 {RER}\| market for |
| Transport by boat | 20,000 km | n.a. | Transport, freight, sea, container ship {GLO}\| market for |

**Washing and sterilisation**

After surgery the reusable drapes and gowns are collected in washing bags and transported by truck to the washing and sterilisation facility. An average transportation distance of 100 km (one-way) is assumed. The reusable drapes and gowns are washed and sterilised. In this process water, electricity and natural gas are consumed. Table SI16 present details on washing and sterilisation processes.

**Table SI16 – Inventory data for the washing process**

| **Flow** | **Amount** | **Unit** | **Remarks/modelling details**  ***Ecoinvent datasets in italics*** |
| --- | --- | --- | --- |
| **Inputs** | | | |
| Transport | 200 | km | Both outward and return transport included. *Transport, freight, lorry 16-32 metric ton, EURO6 {RER}* |
| Electricity | 0.12 | kWh | Dutch electricity mix 2022 ^3^ |
| Natural gas | 0.10 | Nm3 | 1 Nm3 of natural gas combustion is modelled as 32.4 MJ of *Heat, district or industrial, natural gas {Europe without Switzerland}\| heat production, natural gas, at industrial furnace low-NOx >100kW* |
| Water | 12.5 | kg | *Tap water {Europe without Switzerland}\| market for* |
| Detergent | 8.3 | g | *Soap {GLO}\| market for* |
| **Outputs** | | | |
| Waste water | 12.5 | kg | *Wastewater, average {Europe without Switzerland}\| treatment of* |

Some of the reusable drapes and gowns have an adhesive edge. This adhesive edge is created by applying a wash-soluble adhesive tape after each washing cycle and before sterilisation. The adhesive tape consists of two pressure-sensitive hotmelt adhesive layers on a tissue carrier, with a paper release liner on top. Table SI17 provides the modelling details for the adhesive edge. The amounts of material and electricity used in the production process have been provided by the producer of the adhesive tape, but are confidential and therefore not reported here. The tissue carrier is very thin compared to the other layers and is therefore assumed to be negligible. Waste treatment of the adhesive tape is not included in the model. This would only concern the release liner, as the adhesive layers and tissue carrier dissolve during the washing process. As the release liner is made of paper and the weight is relatively small compared to the weight of the reusable drapes and gowns it is used on, the environmental impact of the waste treatment of the release liner is assumed to be negligible.

**Table SI17 – Inventory data for the adhesive edge**

| **Input** | **Remarks/modelling details**  ***Ecoinvent datasets in italics*** |
| --- | --- |
| Release liner | Kraft paper {RER}\| market for |
| Adhesive layers | Butyl acrylate {RER}\| market for |
| Tissue carrier | Not included in the model, very thin layer compared to other layers, environmental impact assumed to be negligible. |
| Electricity | Dutch electricity mix 2022 ^3^ |

**End-of-life**

All products, both disposable and reusable, are assumed to be incinerated at end-of-life. In the baseline analysis, incineration without energy recovery is used, as this is the case for hospital waste in the Netherlands. The emissions from the waste incineration process are modelled using the most relevant Ecoinvent dataset for each material included in the model. For most materials, appropriate datasets/proxies are available. However, for ePTFE laminate the incineration of PET is used as a proxy, which does not include any possible emissions of fluorine compounds from incineration of ePTFE. A transport distance of 100 km by truck to waste incineration is assumed. Table SI18 provides insights into the full details of the end-of-life processes.

**Table SI18 – Modelling details of end-of-life processes**

| **Type of material** | ***Ecoinvent dataset*** |
| --- | --- |
| HDPE, LDPE, sticker | Waste polyethylene {CH}\| treatment of waste polyethylene, municipal incineration FAE |
| PET, polyester microfiber, ePTFE laminate, finish | Waste polyethylene terephthalate {CH}\| treatment of waste polyethylene terephthalate, municipal incineration FAE |
| PVC | Waste polyvinylchloride {CH}\| treatment of waste polyvinylchloride, municipal incineration FAE |
| PP | Waste polypropylene {CH}\| treatment of waste polypropylene, municipal incineration FAE |
| Velcro, silicone, PEVA | Waste plastic, mixture {CH}\| treatment of waste plastic, mixture, municipal incineration FAE |
| Iron, aluminium | Scrap aluminium {CH}\| treatment of scrap aluminium, municipal incineration FAE |
| Cotton | Waste textile, soiled {CH}\| treatment of waste textile, soiled, municipal incineration FAE |
| Paper | Waste graphical paper {CH}\| treatment of waste graphical paper, municipal incineration FAE |
| Rubber, elastic | Waste rubber, unspecified {CH}\| treatment of waste rubber, unspecified, municipal incineration FAE |
| Glue | Waste paint {CH}\| treatment of waste paint, municipal incineration FAE |

**S.I. 4: Modelling details sensitivity analyses**

*Electricity mix*

In the sensitivity analysis studying the effect of the geographical scope, the electricity mix is varied. Table SI19 shows how the electricity mixes of Europe, Sweden and Poland are modelled.

**Table SI19 - Modelling details of the electricity mixes of Europe, Sweden and Poland**

| **Location** | ***Ecoinvent dataset*** |
| --- | --- |
| Europe | Electricity, medium voltage {RER}\| market group for |
| Sweden | Electricity, medium voltage {SE}\| market for |
| Poland | Electricity, medium voltage {PL}\| market for |

*Energy recovery*

A sensitivity analysis is conducted to study the effect of waste incineration with energy recovery. The energy recovery efficiencies are provided in Table SI20. The recovered energy replaces the average national electricity mix (see Table SI19) and heat from natural gas (ecoinvent dataset: “*Heat, district or industrial, natural gas {Europe without Switzerland}| market for*”). Lower heating values (LHVs) of the materials are provided in table SI21. These LHVs are based on the information provided in the ecoinvent datasets of the materials.

**Table SI20 - Energy recovery efficiencies of MSWIs**

|  | **Thermal efficiency** | **Electric efficiency** | **Source** |
| --- | --- | --- | --- |
| The Netherlands | 31% | 18% | Stichting Nationale Milieudatabase^4^ |
| Europe | 35% | 15% | CEWEP^5^ |
| Sweden | 73% | 11% | CEWEP^5^ |
| Poland | 28% | 15% | CEWEP^5^ |

**Table SI21 - Lower heating values (LHVs) of all materials included in the model**

| **Material** | **LHV (kg/MJ)** |
| --- | --- |
| HDPE | 42.5 |
| LDPE | 42.5 |
| PVC | 21.5 |
| PP | 32.8 |
| PET | 23 |
| Velcro | 30.9 |
| Silicone | 9.8 |
| Iron | 0 |
| Aluminium | 0 |
| Cotton | 13 |
| Paper | 10.2 |
| Rubber | 26.4 |
| Elastic | 26.4 (modelled as rubber) |
| Sticker | 42.5 (modelled as LDPE) |
| PEVA | 36 |
| Glue | 18.2 |
| Finish | 23 (modelled as PET) |

**Supplementary Results**

**S.I. 5: Results break-even analysis reuse rate**

Table SI22 presents the break-even reuse rates for drapes and gowns at which reusable products begin to have a lower environmental impact than disposable ones.

**Table SI22 - Results break-even analysis reuse rate**

| Environmental impact indicator | Reuse rate of reusable drapes and gowns below which the disposable tray has a lower impact than the hybrid tray |
| --- | --- |
| Global warming | 16 |
| Stratospheric ozone depletion | 1757 |
| Ionizing radiation | 10 |
| Ozone formation, Human health | 3 |
| Fine particulate matter formation | 5 |
| Ozone formation, Terrestrial ecosystems | 3 |
| Terrestrial acidification | 5 |
| Freshwater eutrophication | 5 |
| Marine eutrophication | No break-even point |
| Terrestrial ecotoxicity | 5 |
| Freshwater ecotoxicity | 5 |
| Marine ecotoxicity | 5 |
| Human carcinogenic toxicity | 6 |
| Human non-carcinogenic toxicity | 6 |
| Land use | No break-even point |
| Mineral resource scarcity | 11 |
| Fossil resource scarcity | 4 |
| Water consumption | 5 |

**References**

1. Weidema, B. P. and M. S. Wesnæs (1996). "Data quality management for life cycle inventories—an example of using data quality indicators." Journal of cleaner production **4**(3-4): 167–174.

2. Martí, M., C. Alonso and A. Manich (2019). P041_0614_ Mitigation of environmental impact caused by DWOR textile finishing chemicals studying their nontoxic alternatives. Proceedings of the 19th World Textile Conference-Autex 2019.

3. Maarten Bruinsma, M. N. (2023). Ketenemissies elektriciteit. Actualisatie elektriciteitsmix 2021. CE Delft, CE Delft.

4. Milieudatabase, S. N. (2025). Bepalingsmethode milieuprestatie bouwwerken, NMD.

5. Reimann, D. (2012). "CEWEP energy report III." Scientific & Technical Advisor to CEWEP, Bamberg Dezember.
